# Supplementary material for: Clinical and molecular epidemiology of influenza viruses from Romanian patients hospitalized during the 2019/20 season
Source: PLoS One. 2021 Nov 12;16(11):e0258798. doi: 10.1371/journal.pone.0258798 (PMC8589178; doi:10.1371/journal.pone.0258798)
Supplement: S1 Table — (DOCX) [file pone.0258798.s001.docx]

| Characteristics | Influenza A,  n=272 | Influenza B,  n=242 | Statistical analysis |
| --- | --- | --- | --- |
| Male gender, n (%) | 150 (55.1%) | 129 (53.3%) | p=0.743 |
| Median age, years (IQR) | 5.8 (2.2, 29.8) | 6.3 (2.1, 12.5) | p=0.137 |
| Age groups |  |  |  |
| Infants, <1 year, n (%) | 40 (14.7%) | 31 (12.8%) | p=0.532 |
| Toddlers, 1-2 years, n (%) | 42 (15.44%) | 50 (20.66%) | p=0.123 |
| Preschoolers, 3-4 years, n (%) | 44 (16.17%) | 27 (11.15%) | p=0.099 |
| School children, 5-13 years, n (%) | 57 (20.95%) | 79 (32.64%) | p=0.002, OR=0.6, 95%CI:0.4-0.8 |
| Teenagers, 14-17 years, n (%) | 11 (4.04%) | 11 (4.54%) | p=0.777 |
| Adults, 18-64 years, n (%) | 55 (20.22%) | 38 (15.7%) | p=0.184 |
| Elderly adults, ≥65 years, n (%) | 23 (8.45%) | 6 (2.47%) | p=0.003, OR=3.6, 95%CI:1.4-9.0 |
| Clinical characteristics |  |  |  |
| Fever, n (%) | 268 (98.52%) | 236 (97.52%) | p=0.828 |
| Malaise, n (%) | 227 (83.45%) | 204 (84.29%) | p=0.657 |
| Headache, n (%) | 123 (45.22%) | 111 (45.86%) | p=0.824 |
| Myalgia, n (%) | 92 (33.82%) | 82 (33.88%) | p=0.941 |
| Cough, n (%) | 259 (95.22%) | 233 (96.28%) | p=0.345 |
| Sore throat, n (%) | 121 (44.48%) | 120 (49.58%) | p=0.218 |
| Dyspnea, n (%) | 44 (16.17%) | 28 (11.57%) | p=0.142 |
| Nasal congestion, n (%) | 222 (81.61%) | 208(85.95%) | p=0.134 |
| Deterioration, n (%) | 104 (38.23%) | 94 (38.84%) | p=0.934 |
| Chronic conditions, n (%) | 80 (29.4%) | 43 (17.8%) | p=0.002, OR=1.9, 95%CI:1.3-2.9 |
| Cardiovascular disease, n (%) | 40 (14.7%) | 8 (3.30%) | p<0.001, OR=5.0, 95%CI:2.3-10.9 |
| COPD, n (%) | 2 (0.73%) | 0 | p=0.183 |
| Asthma, n (%) | 5 (1.83%) | 4 (1.65%) | p=0.882 |
| Diabetes mellitus, n (%) | 15 (5.51%) | 5 (2.06%) | p=0.045, OR=2.7, 95%CI:1.0-7.7 |
| Renal impairment, n (%) | 7 (2.57%) | 3 (1.23%) | p=0.277 |
| Rheumatologic disease, n (%) | 10 (3.67%) | 1 (0.41%) | p=0.011, OR=9.1, 95%CI:1.2-71.8 |
| Neurological disease, n (%) | 17 (6.25%) | 10 (4.13%) | p=0.291 |
| Liver disease, n (%) | 11 (4.04%) | 5 (2.06%) | p=0.203 |
| Neoplasm, n (%) | 7 (2.57%) | 5 (2.06%) | p=0.713 |
| Obesity, n (%) | 15 (5.51%) | 7 (2.89%) | p=0.147 |
| HIV infection, n (%) | 5 (1.83%) | 9 (3.71%) | p=0.882 |
| Days of hospitalization,  median (IQR) | 5 (3, 6) | 5 (3, 6) | p=0.934 |
| Respiratory failure with supplemental oxygen, n (%) | 32 (11.76%) | 20 (8.26%) | p=0.199 |
| ICU admission, n (%) | 11 (4.04%) | 10 (4.13%) | p=0.946 |
| Deaths, n (%) | 3 (1.10%) | 0 | p=0.103 |

COPD - chronic obstructive pulmonary disease, ICU – intensive care unit
